# Closed-loop for type 1 diabetes – an introduction and appraisal for the generalist

**DOI:** 10.1186/s12916-017-0794-8

**Published:** 2017-01-23

**Authors:** Lia Bally, Hood Thabit, Roman Hovorka

**Affiliations:** 1 University of Cambridge Metabolic Research Laboratories and NIHR Cambridge Biomedical Research Centre, Wellcome Trust-MRC Institute of Metabolic Science, Box 289, Addenbrooke’s Hospital, Hills Road, Cambridge, CB2 0QQ UK; 2 Department of Diabetes & Endocrinology, Cambridge University Hospitals NHS Foundation Trust, Cambridge, UK; 3 Department of Diabetes, Endocrinology, Clinical Nutrition & Metabolism, Inselspital, Bern University Hospital, University of Bern, Bern, Switzerland; 4 Department of Paediatrics, University of Cambridge, Cambridge, UK

**Keywords:** Type 1 diabetes, Artificial pancreas, Closed-loop, Glucose control

## Abstract

**Background:**

Rapid progress over the past decade has been made with the development of the ‘Artificial Pancreas’, also known as the closed-loop system, which emulates the feedback glucose-responsive functionality of the pancreatic beta cell. The recent FDA approval of the first hybrid closed-loop system makes the Artificial Pancreas a realistic therapeutic option for people with type 1 diabetes. In anticipation of its advent into clinical care, we provide a primer and appraisal of this novel therapeutic approach in type 1 diabetes for healthcare professionals and non-specialists in the field.

**Discussion:**

Randomised clinical studies in outpatient and home settings have shown improved glycaemic outcomes, reduced risk of hypoglycaemia and positive user attitudes. User input and interaction with existing closed-loop systems, however, are still required. Therefore, management of user expectations, as well as training and support by healthcare providers are key to ensure optimal uptake, satisfaction and acceptance of the technology. An overview of closed-loop technology and its clinical implications are discussed, complemented by our extensive hands-on experience with closed-loop system use during free daily living.

**Conclusions:**

The introduction of the artificial pancreas into clinical practice represents a milestone towards the goal of improving the care of people with type 1 diabetes. There remains a need to understand the impact of user interaction with the technology, and its implication on current diabetes management and care.

## Background

Glycaemic control with insulin therapy is influenced by factors such as insulin dosage, absorption and timing [1], as well as physiological and lifestyle factors such as physical activity, meal intake, hormones and illness [2–4]. These factors may contribute to the notable variability in insulin requirements, which makes self-management of type 1 diabetes challenging [5]. As a result, the majority of people with type 1 diabetes are still unable to achieve their recommended therapeutic goals. Therefore, there is currently an unmet need to improve glycaemic control and alleviate the risk of hypoglycaemia whilst reducing the burden of type 1 diabetes self-management.

## Currently available technology

The use of continuous glucose monitoring in motivated individuals has shown improvements in glycaemic control [6] and complements insulin pump management in the form of sensor-augmented pump therapy [7]. Advanced features of sensor-augmented pump therapy have recently been introduced into clinical practice; this includes automatic suspension of insulin delivery when a pre-set glucose threshold is reached (low glucose suspend) [8] or predicted to be reached (predictive low glucose suspend) [9]. Both approaches have shown significant reduction in the risk and burden of hypoglycaemia, particularly in hypoglycaemia-prone individuals, with the latter stabilising glucose levels perhaps due to a reduced need for supplemental carbohydrates to treat hypoglycaemia often resulting in rebound hyperglycaemia [9].

## Foundation of the artificial pancreas

**Fig. 1:**
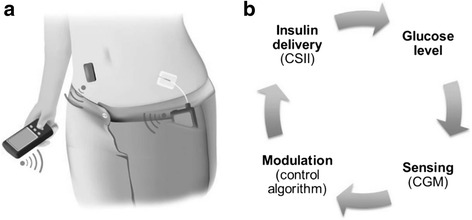
**a** A closed-loop system comprising a glucose sensor (black rectangle on the left-hand side of the abdomen), an insulin pump (device in the pocket), and a mobile-sized device containing the control algorithm (in patient’s hand). Each component communicates with the other wirelessly (adapted from Hovorka [32]). **b** The closed-loop system mimics the physiological feedback normally provided by the pancreatic beta cell

Parallel to these relatively simple but progressive approaches to mitigate the risk of hypoglycaemia, bolder developments have taken place to link insulin delivery directly to glucose levels. Closed-loop insulin delivery, also known as the Artificial Pancreas, differs from conventional pump therapy and low glucose management technology through a control algorithm that autonomously increases and decreases subcutaneous insulin delivery based on real-time sensor glucose levels (Fig. 1) [10]. Closed-loop systems can be categorised into insulin-only and bi-hormonal control systems [11]. The former achieve target glucose level by modulating insulin delivery alone, whereas the latter utilise both insulin and glucagon. Manual meal-time announcement and prandial insulin boluses are still recommended to be carried out by the user to overcome the delay in insulin action profile of currently available insulin analogues. This ‘hybrid’ closed-loop approach is in contrast to a ‘fully’ closed-loop approach, in which user input to the control algorithm related to meals is not required. The recent approval of the US Food and Drug Administration (FDA) of a hybrid closed-loop insulin delivery system has been borne by the hope and expectations of type 1 diabetes community, patient advocacy organisations and healthcare providers that it may help reduce the burden of type 1 diabetes management and improve glycaemic control [12]. This signifies a major advance in fulfilling regulatory requirements, enabling people with type 1 diabetes to benefit from this novel technology.

## Clinical evidence behind the Artificial Pancreas

Clinical studies have progressed over the past decade from controlled research facility [13] and supervised transitional settings [14, 15] to free-living home studies [16, 17]. Two recent multicentre randomised crossover free-living home studies evaluated single-hormone hybrid closed-loop system use over a longer period in adults. In one study, 2-month closed-loop use in the evening, after dinner, and overnight was compared to sensor-augmented pump therapy [17]. Closed-loop insulin delivery improved the time spent in the target glucose range between 3.9 and 10.0 mmol/L and HbA1c, and reduced the time spent hypoglycaemic. This was in line with a 3-month study comparing day-and-night closed-loop use with sensor-augmented pump therapy [16], where improvements in time in the target glucose range, HbA1c and hypoglycaemia were also shown. Through the modulation of insulin delivery by closed-loop control algorithm, more consistent glucose levels were achieved without increasing the total amount of insulin delivered. The largest clinical study of a hybrid single hormone closed-loop system to date has demonstrated safety in an outpatient setting over 3 months [18].

Closed-loop application has also been evaluated in other populations. Closed-loop use in children and adolescents has shown improvement in either time spent in the glucose target range [19] or reduction in hypoglycaemia risk [20]. In pregnant women with type 1 diabetes, improvements in time in the glucose target range and mean glucose was achieved by closed-loop without increasing hypoglycaemia risk compared to sensor-augmented pump therapy [21].

A number of research groups are currently focussing on the incremental benefits of adding glucagon in bi-hormonal closed-loop systems to further reduce the residual risk of hypoglycaemia with single-hormone closed-loop systems or to allow more aggressive insulin dosing and use glucagon to counteract a potential insulin overdose [22]. The first study to evaluate the short-term outpatient use of a bi-hormonal closed-loop system in adults with type 1 diabetes applying more aggressive insulin dosing showed lower mean glucose level and hypoglycaemia risk during the 5-day study period [23]. Similar glycaemic benefits using bi-hormonal closed-loop systems were observed in pre-adolescent children in a diabetes camp setting [24]. Head-to-head comparison between single-hormone and bi-hormonal non-aggressive closed-loop systems showed no significant difference in the proportion of time in the glucose target range; however, fewer hypoglycaemic events occurred with the bi-hormonal system [25]. In contrast to single-hormone studies, however, bi-hormonal closed-loop systems have not been evaluated during free-living settings over extended periods. The addition of glucagon imposes further demands and complexity in daily life application and is currently limited by the need to reconstitute glucagon daily and the requirement of a second pump. Ongoing research aims to address these issues.

## Towards commercialisation

**Fig. 2:**
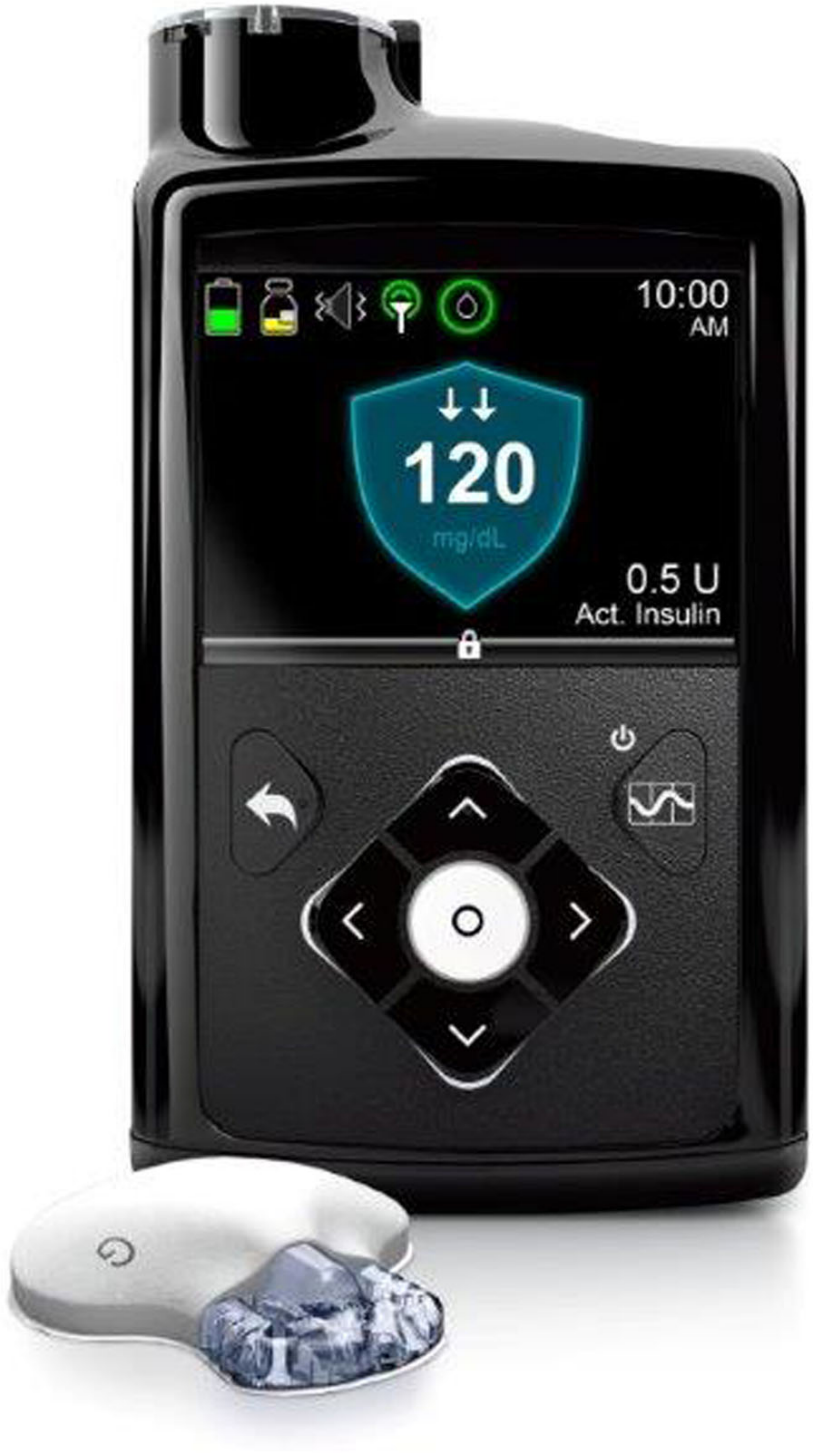
Hybrid closed-loop system comprising fourth generation Enlite 3 glucose sensor, MiniMed® 670G insulin pump, and an integrated proportional-integral-derivative algorithm with insulin feedback (Medtronic, Northridge, CA) (courtesy of Medtronic)

The MiniMed® 670G pump (Medtronic, Northridge, CA), recently approved by the FDA, is a single hormone hybrid closed-loop system with the control algorithm integrated in the insulin pump (Fig. 2). The pump was evaluated in a pivotal clinical trial to assess safety; however, due to the non-randomised study design and lack of a control arm, statements pertaining to its efficacy are limited [18]. The closed-loop system was used day-and-night for 3 months by 94 adults and 30 adolescents. No episodes of severe hypoglycaemia or ketoacidosis were observed. Four serious adverse events, none device-related, were reported.

Other device companies are developing similar single-hormone hybrid closed-loop systems. These include the Bigfoot Smartloop™ system and the Omnipod Artificial Pancreas™ system, both of which are undergoing pilot clinical evaluations (NCT02849288 and NCT02897557, respectively). In addition, commercialisation of a bi-hormonal closed-loop system in Europe is currently being planned with the proposed date of approval in 2017 [26].

## Indication for closed-loop in clinical practice

Criteria for application of closed-loop in clinical practice are yet to be refined. Evidence is being collected in large randomised clinical trials to justify the use of the technology in a wide range population to funders and healthcare professionals [16, 19, 21, 27], as well as elucidating its glycaemia-related and psychosocial impacts. Pathways range from using closed-loop systems in lieu of sensor-augmented pump therapy, to application of closed-loop systems immediately post-presentation of type 1 diabetes if a decline in residual C-peptide secretion could be slowed down. In the longer term, the trend in increasing use of insulin pump therapy could be accelerated and a greater proportion of those utilising multiple daily injection may opt for closed-loop systems. Unwillingness or intolerance to wear a continuous glucose monitoring and/or insulin pump, excludes closed-loop use in clinical practice.

## Expectations and implications in clinical practice

Although commercialisation and clinical availability of closed-loop systems is imminent, the implications of user acceptance and interaction with this novel technology are yet to be fully understood. Based on our extensive experience of home studies, amounting to total closed-loop operation of 3380 days, or 9.3 years, in populations such as children, adolescents, pregnancy, and well- and suboptimally controlled adults, we found that users may have different expectations of closed-loop technology and its role in their care. A ‘one size fits all’ may not be the appropriate model, as different user demands need to be catered for. Some participants suggested having a more simplified or ‘hands-off’ approach, which includes minimal input for prandial insulin boluses to reduce the burden of self-management. Others wished to be more involved in their interaction with the closed-loop system, such as being able to ‘adjust’ the control algorithm to their individual needs, i.e. personalisation of their glucose target range based on time of day, state of health or physical activity. The message from our experience is that the designers of closed-loop systems may need to consider both simplified and sophisticated user-system interaction, while accounting for individual needs and expectations.

Current research prototypes and upcoming commercialised systems require on-going user input and interaction. Hybrid systems require user-triggering of insulin boluses for meals that may not match users’ initial expectations of an ‘artificial pancreas’, thus emphasising the ongoing need for education and training of this novel technology. In previously sensor-naïve individuals transitioning to closed-loop therapy, the availability and use of continuous glucose monitoring may introduce additional need for training by educators. For example, the sensor alarms, miscalibration and overreaction to glucose trends can negatively affect user experience, adherence and outcomes, if not addressed earlier on. The immediate impact on currently available structured education programmes [28] is currently unknown and needs further evaluation. The focus of these programmes may therefore need to account for different strategies and approaches in individuals using closed-loop systems, during meals and exercise, for example. The emphasis on diabetes self-management still needs to be maintained in the event of a closed-loop system not being operational due to unavailable sensor data, for example, and the role for technical support, be it from the manufacturer or healthcare providers, needs to be delineated.

## Outlook

It is anticipated that future closed-loop systems may involve the use of non-calibrated sensors which may further reduce device burden [29]. The advent of faster insulins [30] and availability of stable glucagon [31] may enhance the performance of single-hormone and bi-hormonal closed-loop systems, respectively. Although it is expected that no significant added expenses apart from those related to continuous glucose monitoring may be conferred on the user, longer studies evaluating long-term biomedical and psychosocial outcomes, as well as cost-effectiveness, are needed to justify and provide wider reimbursements for all users.

## Conclusions

Although the ideal situation would be a biological cure for type 1 diabetes, where damaged beta-cells could be replaced with healthy ones and be viable, the interim but fast innovating role of the artificial pancreas might be to act as a ‘bridge’ until that cure is found. The rapid progress from ‘bench to bedside’ in the past decade has made the artificial pancreas a reality, and may hopefully lead towards better care in the management of people with type 1 diabetes in the near future.
